# Support for families at home during childhood cancer treatment: a pilot study with Mr.V the Spaceman, a family-based activities tool

**DOI:** 10.1007/s00520-021-05995-3

**Published:** 2021-02-07

**Authors:** Kelly L. A. van Bindsbergen, Patrizia D’Olivo, Marco C. Rozendaal, Johannes H. M. Merks, Martha A. Grootenhuis

**Affiliations:** 1 Princess Máxima Center for pediatric oncology, Utrecht, The Netherlands; 2 Emma Children’s Hospital, Amsterdam UMC, Amsterdam, The Netherlands; 3 Delft University of Technology, Delft, The Netherlands

**Keywords:** Childhood cancer, Family-centered care, Supportive care, At home, Playful tool, Family activities

## Abstract

**Purpose:**

It is important to support families in dealing with the distress that comes along with the diagnosis and treatment of childhood cancer. Therefore, we developed a playful tool that families can use at home to support their family functioning and safeguard their normal family life. We pilot tested this new tool called Mr.V and describe how families used and evaluated the tool, and how it could be further improved.

**Methods:**

Mr.V is an interactive dispenser that looks like a spaceman and proposes family activities. These activities are suggested by family members themselves and dispensed by the machine at unexpected moments. Mr.V produced data on how it was used, and a questionnaire and a semi-structured interview were used to evaluate the experiences of families and the potential of this tool.

**Results:**

Ten families with a child with cancer between 5 and 9 years old (*M*_age_ = 6.7 years) who were in active treatment (mixed diagnoses) participated (*n* = 47; *n*_patients_ = 10, *n*_siblings_ = 9, *n*_parents_ = 16). All families used Mr.V for multiple days and were very satisfied with the tool regarding its acceptability, feasibility, and potential effectiveness. They also had suggestions on how the tool could be further improved.

**Conclusion:**

Mr.V is an acceptable and feasible tool that can be implemented by families independently at home, regardless of their level of need for support. Mr.V promoted family activities and therefore has the potential to support family functioning and normal family life at home. Future research should further investigate the effectiveness of this tool.

## Introduction

In the Netherlands, about 650 children are diagnosed with cancer every year [1]. This diagnosis and the often lengthy, demanding treatments have a significant impact on the child, as well as the whole family [2]. The shock of a cancer diagnosis, and the burden of treatment and daily caretaking have an impact on family functioning [3–6]. Among the consequences of this impact on the child and the family is a loss of normality [7]. Everyday routines change, family relationships are challenged, and social activities get hampered by the distress that comes along with the disease and its treatment. Therefore, it is important to support families in dealing with this distress, and to safeguard their normal everyday family life.

The Pediatric Psychosocial Preventative Health Model (PPPHM) is a biopsychosocial framework that can be used in assessment and treatment of families of children in pediatric health care settings [8]. According to the PPPHM, all families that are affected by childhood cancer experience some level of distress, and should therefore have access to a certain level of support. This universal support should have a preventative goal and incorporate general interventions or services to assist families [8].

In the Netherlands, various preventative sources of support are available at the hospital for families throughout the treatment of the child. As part of standard care [9], child life specialists are available to prepare and support children during medical procedures to prevent medical traumatic stress. Also, social workers are available to support parents emotionally and help them in continuing their family life and overcoming practical issues. If needed, a team of psychologists is available to provide specialized additional psychosocial care. However, there are limited general preventative interventions or services available to provide all family members with support at home.

An important way of supporting family functioning at home, and maintaining a normal family life, is by supporting family activities or promoting family quality time. According to the Core and Balance Model of Family Leisure Functioning [10], core family leisure includes experiences that are typically home-based, relatively accessible, low-cost, and common. Such activities often require minimal planning and resources, can be spontaneous and informal, and provide a safe, consistent, and typically positive context in which family relationships tend to be enriched and feelings of family closeness increased. Therefore, play is an appropriate way to provide family-centered care [11].

A tool that was developed to support families with a child with cancer at home using a playful approach is the Cellie Cancer Coping Kit [12]. The Cellie Cancer Coping Kit is designed to promote coping and decrease distress in children undergoing cancer treatment, and encourages parents and children to use the tool together. However, the focus of the tool is on the child and not the whole family, and also relies on psycho-education.

To address these gaps in family-centered and home-based supportive care for families with a child with cancer, we developed a playful tool that stimulates family activities. This tool was created in collaboration with design researchers and called Mr.V (short for “Mr. Verrassing”, which translates to “Mr. Surprise” in English). Mr.V is an interactive dispenser that looks like a spaceman and proposes family activities. These activities are suggested by family members themselves and dispensed by the machine at unexpected moments (i.e. as a surprise). The aim of Mr.V is to help families engage in behavior that supports their family functioning and normal family life at home.

The purpose of this pilot study was to describe the experiences of families with this new tool, and to evaluate its potential to support families with a child with cancer during treatment at home. Specifically, our research questions were as follows: (1) How do families with a child with cancer use Mr.V with regard to time and frequency of use? (2) How do families evaluate Mr.V in terms of acceptability, feasibility (ease of use), and potential effectiveness? (3) How do families think Mr.V can be improved?

## Methods

### Description of the prototype of Mr.V: a vending machine

**Fig. 1:**
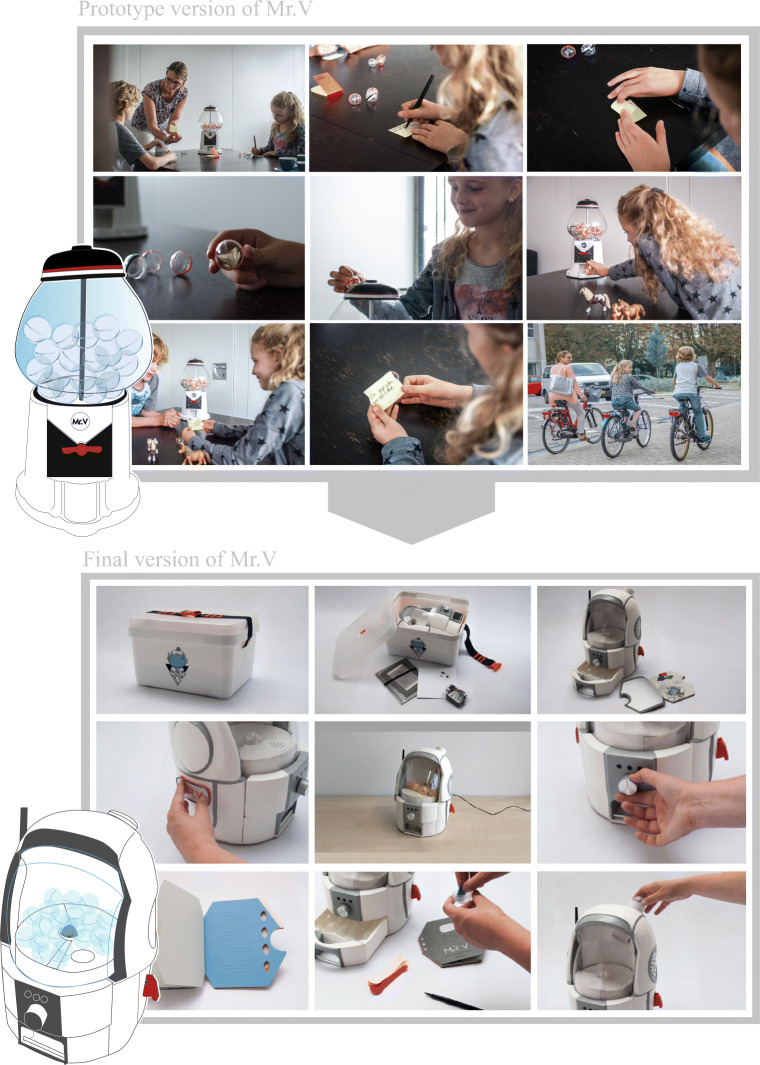
Prototype and final version of Mr.V

We first developed a prototype of Mr.V (Fig. 1). This prototype resembled a gumball vending machine, but dispending surprises instead of gumballs. The surprises were notes written by family members, ranging from activities they would like to do together, to compliments and jokes. The notes were inserted into small plastic balls and stored in the machine. These plastic balls with notes were dispensed by the machine at unexpected moments during the week. Family members could also request a surprise on demand by pressing a button located at the backside of Mr.V. Before dropping a surprise, Mr.V shuffled the balls and made sound effects.

The prototype of Mr.V was pre-piloted by four families with a child with cancer to evaluate its functionality, and whether families were open to use it and positive about the concept. Families received some example surprises, and a diary to keep track of their use of the machine. The detailed results of this study can be found in D’Olivo et al. [13].

### Description of the final Mr.V: a spaceman

**Fig. 2:**
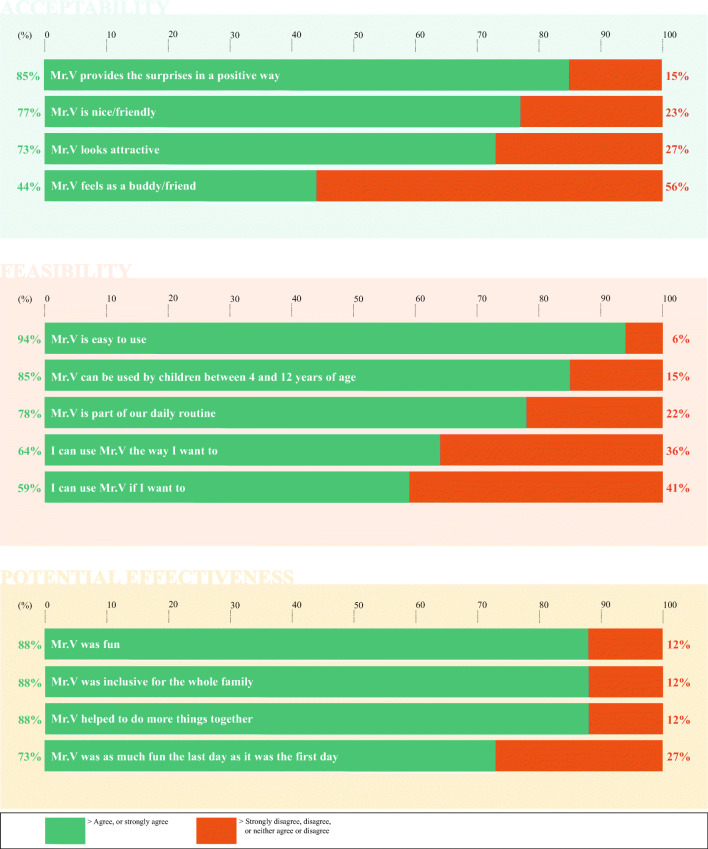
Acceptability, feasibility, and potential effectiveness of Mr.V as reported by the families (ordered from high to low)

Based on the results of this pilot study, changes were made to the prototype creating Mr.V the Spaceman (Fig. 2). Adaptations consisted of a new spaceman look and new features, such as a build-in pen, a build-in drawer, a little booklet, a time knob, and the possibility to collect data on how it is used. Also the sound effects, lights, and button to request surprises on demand were modified.

The antenna on the head of the spaceman was a detachable pen that could be used to write the notes for the surprises. A drawer at the bottom of the machine was added to store the plastic balls when empty, together with a booklet. The booklet contained information about the study, a page where families could establish rules regarding the surprises (i.e. kind of surprises and possible costs of surprises) and colored removable paper stripes to write the notes on. Four different colors were available so that families could make distinctions in the surprises. Colors could for example represent a family member the surprise was from or for, or a type of surprise (i.e. for good or bad days, or for indoors or outdoors). The oxygen hose of the spaceman served as the opening to add the filled plastic balls to the machine. Mr.V was also equipped with an electricity plug, an instruction manual, and a one-page letter about how Mr.V came from space to stay with the family and provide surprises.

To anticipate the surprise dropping moment, the lights in the helmet of Mr.V start to twinkle, and the balls (visible through the helmet) start to shuffle, followed by a sound effect. The button to request a surprise on demand was redesigned as an emergency switch on the side of Mr.V. A time knob was added, with which families could set a preferred time range for obtaining the surprises: either in the morning, afternoon, or evening. During the night, Mr.V turned off automatically, to avoid children getting up at night to check for surprises. Different kinds of sound effects were linked to the time knob, and when Mr.V was turning off and on.

### Participants and procedure

Families were recruited via an information letter handed out by their pediatric oncologist or child life specialist. After 1 week, the families were contacted by telephone by one of the researchers to ask whether they wanted to participate. Inclusion criteria were families with a child who was (1) in active treatment for cancer, (2) not hospitalized, (3) between 4 and 12 years of age, and (4) spoke Dutch sufficiently.

### Procedure

Two copies of Mr.V were available, and the study consisted of three phases. In the introduction phase, Mr.V was presented to the families either at their home or at the hospital. Instructions about the main functions of Mr.V were given, as well as the user manual and booklet. Families were invited to try out Mr.V and to ask questions (±15 min). Next, during the usage phase, families were asked to use Mr.V for at least 1 week at their homes. In the concluding evaluation phase, families were interviewed either at home or at the hospital and filled-out an evaluation questionnaire (±60 min). The study was conducted with permission of the Medical Ethics Committee of the University Medical Center Utrecht in the Netherlands and in accordance with their regulations.

### Measures

#### Machine data

To gain insight into how families used Mr.V, it registered: How many days it was used, how many balls were added to it and when, how many surprises were dropped automatically and at what time of the day, how many times families used the button, time knob, or unplugged Mr.V. Separately, the researchers counted the number of days the families had Mr.V at home, and how many notes were made (i.e. how many paper stripes were taken out of the booklet).

#### Questionnaire

The evaluation questionnaire consisted of statements about Mr.V’s feasibly (ease of use; 5 items), acceptability (4 items), and potential effectiveness (4 items). All statements were rated on a 5-point Likert scale ranging from (1) strongly disagree to (5) strongly agree. Examples of the statements were “Mr.V is easy to use”, “Mr.V provides the surprises in a positive way”, and “Mr.V helped to do more things together”. The questionnaire was filled out by all family members who used Mr.V (±10 min).

#### Interview

Semi-structured group interviews, in which all family members participated together, were performed and recorded to evaluate the experiences of families with Mr.V and to discuss possible improvements (±30 min). The interviewer tried as much as possible to obtain answers from all family members, including younger siblings. Example questions were “Who made the surprises?”, “Did you encounter any difficulties (if so, what difficulties)?”, “Do you think Mr.V was valuable for your family during times of treatment (if so, how exactly)?”, and “Would you recommend Mr.V to other families with a sick child (if so, why)?”.

### Data analysis

Data collected from the machine and from the questionnaire were entered to IBM SPSS Statistics (version 25) and described using descriptive statistics. For the questionnaire data, the 5-point Likert scale was dichotomized into disagree (answers 1 to 3) and agree (answers 4 and 5). Data collected through the interviews were transcribed verbatim and translated into English by two research assistants (R.V. and M.S.). The transcriptions were anonymized and analyzed in ATLAS^tm^ by the second author. The analyzed data was checked by the third author who was not involved in the interviews, and discrepancies were discussed with the first author until consensus was researched. Using content analysis, all the responses from families were marked as statements and clustered in a top-down manner and given a theme [14]. Then, the number of themes was reduced according to their relevance (i.e. small themes with only a few statements were included in larger related themes), and clustered in relation to acceptability, feasibility, potential effectiveness, or improvements.

## Results

Eighteen families with a child with cancer were approached to participate in this study. Eight families declined to participate, because of hospitalization of the child with cancer (*n* = 3), no interest (*n* = 2) or finding it too demanding at this point of treatment (*n* = 3). In total, ten families (55.6%) were included and written consent was obtained from all family members (*n* = 47). The families participated in the pilot study between June and December 2018. The children were between 5 and 9 years of age (*M* = 6.7, *SD* = 1.34), and their diagnoses were mixed. More details about the characteristics of the children and their families can be found in Table 1.

**Table 1:** Patient (*n* = 10) and family member (*n* = 47) characteristics

	*n*	%
Patient characteristics (*n* = 10)		
Age		
5 years	3	30.0
7 years	5	50.0
8 years	1	10.0
9 years	1	10.0
Gender		
Boys	8	80.0
Girls	2	20.0
Diagnosis type		
Leukemia or lymphoma	4	40.0
Brain or central nervous system tumor	4	40.0
Solid tumor	2	20.0
Family member characteristics (*n* = 47)		
Patients	10	21.3
Siblings	16	34.0
Parents	21	44.7

### Use of the tool

On average, the families had Mr.V at home for 12 days, of which they used it 8 days. They made between 8 and 36 notes and added 4 to 97 balls to the machine. The notes were added to the machine on the first day, as well as throughout the week, with the exception of one family who added all the surprises on the first day. The content of the notes (*n* = 168) varied within and between families, but mostly contained indoor family activities (e.g. dance together with mom or dad; roasting marshmallows together; play a game together) or outdoor family activities (e.g. eating out, going to the swimming pool, go for a walk in the forest), but also compliments and/or personal messages to each other (e.g. you are a champion and therefore get a big hug; dad, go for a tour in the cabriolet) and jokes (e.g. get another ball; put make-up on mom and dad; give your dad a face mask and take a picture). Mr.V dropped between 2 and 15 surprises during the time the families used it, with an average of 9 surprises. Most surprises were dropped in the afternoon, which was the preferred time setting of most families. The evening was the least favorite time setting. All families used the time knob at least twice to change the preferred timing of the surprises, as well as the button to obtain surprises on demand. This last button was used on average 37 times per family. The machine was unplugged (i.e. turned off) on average 3 times. More specific data on how Mr.V was used by each family can be found in Table 2.

**Table 2:** Use of Mr.V by each family (*n* = 10)

	Families										Statistics		
	1	2	3	4	5	6	7	8	9	10	*M*	SD	Range
Availability													
Mr.V at home (days)	10	8	21	29	11	12	9	7	7	7	12.1	7.3	(7–29)
Mr.V used (days)	4	7	13	15	8	10	8	7	6	4	8.2	3.6	(4–15)
Preparation													
Notes made	10	18	11	8	17	24	36	15	9	20	16.8	8.5	(8–36)
Balls added to machine	4	38	49	31	24	97	56	13	12	26	35.0	27.2	(4–97)
On the first day	1	22	31	8	23	17	4	13	10	13	14.2	9.2	(1–31)
Later days	3	16	18	23	1	80	52	0	2	13	20.8	26.0	(0–80)
Machine actions													
Surprises dropped automatically	2	6	14	15	13	10	11	10	8	4	9.3	4.3	(2–15)
Morning	2	6	0	11	7	1	5	2	0	2	3.6	3.6	(0–11)
Afternoon	0	0	6	4	5	7	3	4	7	2	3.8	2.6	(0–7)
Evening	0	0	8	0	1	2	3	4	1	0	1.9	2.6	(0–8)
Family actions													
Time knob used	2	12	51	57	44	10	63	12	16	16	28.3	22.7	(2–63)
Button used	4	37	34	15	18	87	51	4	12	10	27.2	26.1	(4–87)
Unplugged	3	4	8	7	1	1	0	1	1	4	3.0	2.8	(0–8)

### Evaluation of the tool

The questionnaires were filled out by 35 family members (*n* = 10 patients, *n* = 9 siblings, and *n* = 16 parents) and 31 family members were interviewed (*n* = 10 patients, *n* = 6 siblings, and *n* = 15 parents). An overview of the questionnaire ratings can be found in Fig. 2. These results, together with the 1055 statements of the families that were collected from the interviews, will be described below with regard to feasibility, acceptability, and potential effectiveness. Also, the statements about possible improvements will be described. Due to the richness of the data, only the three most mentioned themes for feasibility, acceptability, potential effectiveness, and improvements are presented here. More details about other themes that emerged can be found in Table 3 (for feasibility, acceptability, and potential effectiveness) and Table 4 (for improvements).

**Table 3:** Interview statements (*n* = 932) about acceptability, feasibility, and potential effectiveness of Mr.V

Themes	Statements (*n*)
Acceptability	*240*
Acceptable	*190*
Liked the functions or interactions or design	102
Positive associations with the tool	48
Purpose was understandable	40
Less acceptable	*50*
Functions or interactions or design could be improved	50
Feasibility	*421*
Feasible	*379*
How they used the tool	86
Appropriate for all family members and others involved	85
Types or amount of surprises they made	60
Appropriate in home context or sensitive setting or hospital	49
Openness in how to use or control the tool	38
Strategies to make surprises or rules about the content	33
Easy to incorporate into family routines or during difficult times	28
Less feasible	*42*
Situations when the tool was overwhelming or less feasible to use	26
Less appropriate features of the tool	16
Potential effectiveness	*271*
Potentially effective	*264*
Provided a positive, fun or exciting experience	94
Valuable for improving family cohesion or interaction	49
Wanted to use it longer for longer lasting effects	37
Involvement of siblings	33
Buddy for children	26
Supportive for parents	25
Potentially less effective	*7*

Numbers in italics are total scores

The themes are ordered from most statements to least statements.

**Table 4:** Interview statements (*n* = 123) about improvements of Mr.V

Themes	Statements (*n*)
Acceptability	*37*
More controllable	*25*
Frequency surprises	9
Parental control	7
Content surprises	5
Fitting family schedule	4
More family-centered	*12*
More inclusive for siblings and older children	9
More child appropriate	3
Feasibility	*46*
Better interaction	*28*
Add humanoid voice with feedback	12
Add more possibilities for interaction	10
Add sound switch/timer	6
Better looks	*18*
Possibility to customize appearance	11
More colors	7
Potential effectiveness	*40*
In other environments	*27*
During treatment	14
In the hospital	10
In other environments	3
More focus on purpose	*13*
Suggestions for best practice to use	7
More guidance for surprises content	4
Purpose more understandable for children	2

Numbers in italics are total scores

The themes are ordered from most statements to least statements.

#### Acceptability

According to the questionnaire ratings, almost all families agree or strongly agree that Mr.V provides the surprises in a positive way. Around three-quarter of the families also agree or strongly agree that Mr.V is nice or friendly and looks attractive. A minority of the families agrees or strongly agrees that Mr.V feels as a buddy or friend. During the interviews, 240 statements were made by family member about acceptability. Most of these statements (79.2%) indicated that the families thought Mr.V was very acceptable. Families talked most about how they liked functions or interactions or design of Mr.V, the positive associations they had with Mr.V, and how the purpose of Mr.V was understandable. Some illustrative examples of some of these statements were as follows: “I liked most of the sounds, they were a bit sparkling, a bit fairytale-like, magic-like”; “It looks nice, it is funny, it is comparable to a gumball machine that we used to have in the past, everyone wanted those”; “It is a sort of a reward system so to say […], there are balls inside with some nice assignments or compliments and once in a while a ball drops”. Some statements (20.8%) were made about features of Mr.V that demonstrated lower acceptability. These statements were mostly on how the functions or interactions with Mr.V could be improved, and on how the design could be improved. Some illustrative examples were as follows: “Only the drawer was not working smoothly, it got stuck a few times”; “I think it can be smaller and made of plastic, it feels a bit heavy now”.

#### Feasibility

According to the questionnaire ratings, almost all families agree or strongly agree that Mr.V is easy to use and can be used by children between 4 and 12 years of age. More than three-quarter of the families agree or strongly agree that Mr.V is part of their daily routine, and more than half of the families agree or strongly agree that they can use Mr.V the way they want to, and if they want to. In the interviews, the families made 421 statements about the feasibility of Mr.V. The majority of these statements (90.0%) indicated that the families thought it was very feasible to use Mr.V. Families mostly explained how they used Mr.V, how the machine was appropriate for all family members and others involved, and the types or amount of surprises they made. Some illustrative examples of some of these statements were as follows: “Most of the times, me and my husband wrote the surprises and then [the child] and his brother, and my daughter opened them”; “Everybody liked it, the youngest two found it most exciting, the oldest one mainly made the assignments, she liked to do that”; “We provided them with some rules like you can ask some presents, but think about more fun things to do, I think that was the goal, how can you do things with the family”. A few statements (10.0%) were made about less feasible features of Mr.V. These statements included situations when Mr.V was overwhelming or less appropriate to use, and features of Mr.V that were sometimes less appropriate. Some illustrative examples were: “I think that Mr.V is a lot of fun, but the frequency of balls, when you would have it for a longer time at home, should not be two surprises per day, that is not doable. Of course, it depends on what kind of surprises you write down, but it is almost not possible to immediately do the things that we had written down”; “Well, I really missed a volume button, the sound was too loud”.

#### Potential effectiveness

According to the questionnaire ratings, almost all families agree or strongly agree that Mr.V was fun, inclusive for the whole family, and helped to do more things together. Around three-quarter of the families also agree or strongly agree that Mr.V was as much fun the last day as it was the first day. In the interviews, the families made 271 statements about the potential effectiveness of Mr.V. Almost all of these statements (97.4%) indicated that the families thought Mr.V could be very effective for them. Families mostly explained how Mr.V provided them with a positive and fun experience, how Mr.V was valuable for improving family cohesion or interaction, and how they wanted to use Mr.V for a longer period of time. Some illustrative examples of some of these statements were as follows: “What comes out [of Mr.V] is always a bit of a surprise, so it is really exciting over and over again and that makes it fun”; “[Mr.V] ‘forces’ you a bit to think about what you can do with the family”; “It remains fun, because the surprises are different every day”. Few statements (2.6%) were made about why Mr.V was potentially less effective. For example: “It disturbs sometimes, that is a point of discussion, on the one hand you want to activate to do family things, but on the other hand I have a 60/70 hours job”.

### Improvements for the tool

In the interviews, families made 123 statements about possibilities to improve Mr.V. Families suggested that the acceptability of Mr.V would be higher if Mr.V would be more controllable, and even more family-centered. The feasibility to use Mr.V could be improved in terms of the interaction with the tool, and by giving Mr.V better looks. The potential effectiveness could be enlarged by also using Mr.V in other environments, and by putting more focus on its purpose. Examples of this were as follows: “It would be nice if I was able to change the setting of when Mr.V goes to sleep”; “When it would maybe become available in the shops, I would like to choose my own color”; “I think it is nice to provide the parents with some tips about what to write on the notes”; “I certainly see the potential for the market and for schools, people who work with rewarding systems or want to connect, team-building kind of things”.

## Discussion

The objective of this pilot study was to describe the experiences of families with a newly developed tool called Mr.V, and to evaluate the potential of this tool to support family functioning and normal family life at home during cancer treatment by promoting family activities. We found that all families used Mr.V for multiple days, regardless of differences in family composition, the diagnosis of the child, or the child’s age. There were many variations noticeable between families in how they used the machine. More specifically, in how they prepared the surprises, how much they let Mr.V act on its own, and how intensively they used the functions of the tool. Therefore, we speculate that families were able to use Mr.V in their own way, and adapt it to their own preferences and routines, providing evidence of its universal applicability.

In their evaluation of Mr.V, families were overall very satisfied with the tool. In line with responses to another healthcare tool to promote coping and decrease distress in children undergoing cancer treatment [12], we found that Mr.V was easy and fun to use, well designed, and provided a relevant and positive experience. We also found that Mr.V was inclusive and appropriate for the whole family, helped families to do more things together, and improved family cohesion and interaction. However, Mr.V was not considered as a buddy or friend. Families proposed to make Mr.V more interactive (e.g. add a voice and make it more responsive), which is in line with research on social robots in healthcare that have these qualities and are considered as companions [15]. Families also suggested to not only or exclusively use Mr.V at home but also in other environments, such as the hospital.

### Clinical implications

The development of Mr.V would not have been possible without the valuable collaboration with design researchers. This collaboration is an example of how design can contribute to innovations in healthcare. Design researchers are able to translate needs and ideas of families into directions where it is possible to intervene and to shape new ways of care [16]. They are able to design and develop new technologies and medical devices that promote health in new, different, more appealing, and playful ways [17].

### Limitations and future research

Mr.V was tested by families during a relatively short period of time, and it would be interesting to find out how the tool would be used and could promote family activities throughout the entire period of cancer treatment. Mr.V should also be tested more, to establish its effectiveness in supporting families during childhood cancer treatment. Measures on feelings of normality, feelings of support, empowerment, resilience, and feelings of distress could help to evaluate how meaningful Mr.V is for families.

Even though we designed Mr.V as a preventative tool to generally support families, it may also be useful as an intervention for specific families that are at elevated risk for distress by providing therapeutic messages. The advantages of using Mr.V for this are that the assignments are provided in a fun and more appealing way (i.e. makes it feel less therapeutic), and that families can be reminded of the assignments throughout the week in a playful way. Likewise, it could be investigated whether Mr.V would also be applicable to families dealing with other kinds of illnesses or distress, or for children with special needs.

It is important to realize that further financial support is needed to re-design Mr.V into a more advanced version, following the suggestions provided by families. This new version of Mr.V should resemble a commercial product, should be easy to program according to the needs of each family, and should be more responsive in line with the new trends of social robots for children [18]. However, next to financial support for re-designing, there will be costs involved for hospitals to purchase the tool. Although the tool is not very complex and should therefore be affordable to produce, hospitals could also select specific families who will benefit more from Mr.V to reduce the number of purchases. Additionally, hospitals will need to develop a service system to distribute the tool, which could be in collaboration with for example family organizations that are connected to the hospital.

## Conclusion

Mr.V is a promising family-centered tool for families dealing with childhood cancer that provides supportive care at home in addition to standard care that is available at the hospital. Mr.V is an acceptable and feasible tool that can be implemented by families independently at home, regardless of their level of need for support. Mr.V promotes family activities, and therefore has the potential to support family functioning and normal family life at home. However, more research on the effectiveness of Mr.V is needed.

## Data Availability

The authors have full control of all primary data and allow the journal to review the data and material if requested.
